# Aphid Diversity in Canadian Amber: Description of New Taxa †

**DOI:** 10.3390/insects17070750

**Published:** 2026-07-22

**Authors:** Bartosz Ogłaza, Małgorzata Kalandyk-Kołodziejczyk, Piotr Wegierek

**Affiliations:** Institute of Biology, Biotechnology and Environmental Protection, Faculty of Natural Sciences, University of Silesia in Katowice, Bankowa 9, 40-007 Katowice, Poland; bartosz.oglaza@us.edu.pl

**Keywords:** aphids, taxonomy, systematics, amber inclusions, Canadian amber

## Abstract

Canadian amber, dated to the late Campanian (79–78 Ma), is the youngest known Late Cretaceous amber deposit containing insect inclusions. Aphids represent one of the most abundant insect groups preserved in this amber. In this study, we describe an apterous morph of *Alloambria infelicis* Kania & Wegierek, 2005 and three new species from Canadian amber: *Matronaphis breviantennata*, *Curraphis velox*, and *Latialiaphis oblita*. Only the latter species is described based on an alate morph. We also analysed the holotypes of the following species: *Ambaraphis kotejai* Kania & Wegierek, 2005, *Ambaraphis costalis* Richards, 1966, *Alloambria infelicis*, *Alloambria caudata* Richards, 1966 and *Pseudambria longirostris* Richards, 1966. Apterous morphs reported from Late Cretaceous deposits share several morphological features with extant aphids, including appendage structures (antennae with distinct processus terminalis and cauda) and other characteristics such as single secondary rhinaria and abdominal wax glands. Our results expand the known morphological diversity of aphids in Canadian amber.

## Simple Summary

In this study, three Late Cretaceous fossil aphid species from Canadian amber are described. We also describe an apterous morph of *Alloambria infelicis* and examine holotype specimens of the species known from this resin. Our research expands the knowledge of aphid diversity during the Late Cretaceous.

## 1. Introduction

The first scientific reports on Canadian amber appeared at the end of the 19th century in the context of its commercial use [1,2]. It was soon recognised that the amber contained abundant animal inclusions [3]. However, it was not until the 1960s that these inclusions regained attention and were again subjected to systematic study. At that time, more than 30 new locations, similar to the Cedar Lake site (Manitoba, Canada), had also been reported [4]. The deposits date from the late Campanian (79–78 Ma) [5], which means that Canadian amber is the youngest Cretaceous fossil resin containing diverse insects [6,7]. *Parataxodium* Arnold and Lowther (Cupressaceae) was most likely the main producer of the resin in question [6,8]. Descriptions of approximately 124 species (mainly insects) from Canadian amber have been published to date; they represent 10 orders and 54 families [6]. Aphids (mainly nymphs) are one of the most abundant groups, constituting over one-third of its insect species [9].

**Figure 1:**
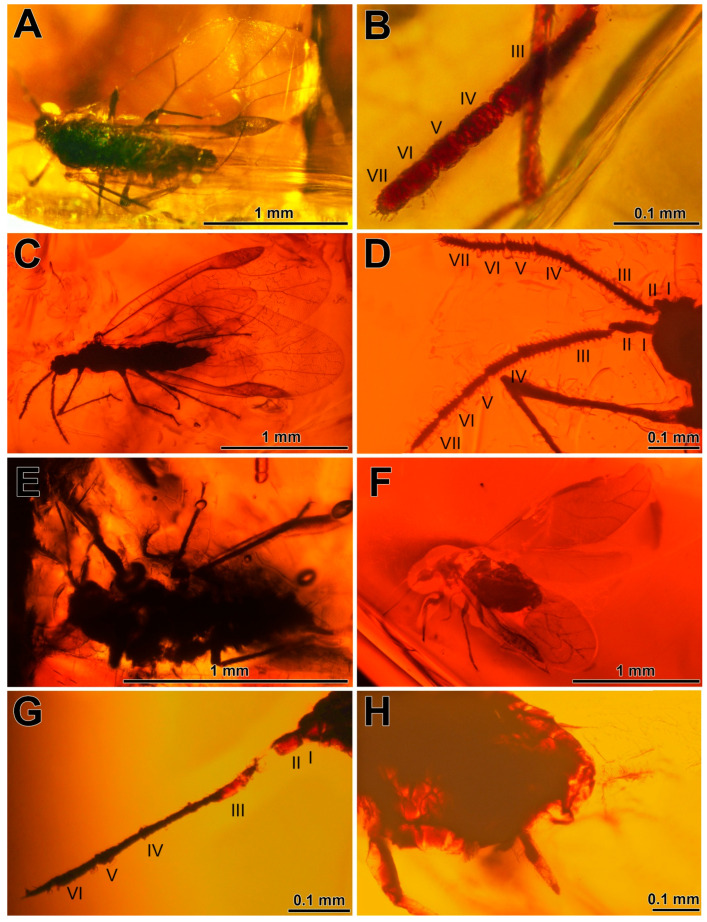
*Palaeoaphis archimedia* holotype: (**A**) dorsal side of the body, (**B**) left antenna; *Ambaraphis costalis* holotype: (**C**) ventral side of the body (fluorescence and phase-contrast microscopy), (**D**) antennae (fluorescence and phase-contrast microscopy); *Alloambria caudata* holotype: (**E**) ventral side of the body (fluorescence and phase-contrast microscopy); *Pseudambria longirostris* holotype: (**F**) lateral side of the body (fluorescence and phase-contrast microscopy), (**G**) left antenna (phase-contrast microscopy), (**H**) posterior part of abdomen seen from the left side (phase-contrast microscopy). Abbreviations: I–VII antennal segments.

*Canadaphis carpenteri* Essig, 1937 was the first aphid species described from Canadian amber [3]. The first publication devoted exclusively to aphids was by Richards [10] and reported five new genera and species. However, photographic documentation of most of these taxa is presented here for the first time (Figure 1).

Professor O.E. Heie made a major contribution to the study of aphids in Canadian amber, describing 13 species [11,12,13,14,15]. In addition, two further publications [16,17] describing new morphs and species from Canadian amber are available.

Before this study, representatives of six families had been described from Canadian amber: Canadaphididae [*Canadaphis* (one sp.); *Alloambria* (two sp.), *Pseudambria* (one sp.)] [10,11,15,16,17]; Cretamyzidae [*Cretamyzus* (one sp.)], [14]; Drepanosiphidae (*Aniferella* (one sp.)] [10]; Mesozoicaphididae [*Albertaphis* (one sp.), *Calgariaphis* (one sp.), *Campaniaphis* (one sp.), *Mesozoicaphis* (four sp.)], [14]; Palaeoaphididae [*Ambaraphis* (two sp.), *Longiradius* (one sp.), *Palaeoaphidiella* (one sp.), *Palaeoaphis* (one sp.)] and Tajmyraphididae [*Grassyaphis* (one sp.)] [10,11,12,13,16]. Additionally, two genera [*Canaphis* (one sp.) and *Aphidinius* (one sp.)] not assigned to any family are also described.

Most of the taxa described so far, unlike the modern fauna, are classified as “aphid ovipara groups”; only Canadaphididae, Drepanosiphidae, and Cretamyzidae are classified as “aphid vivipara”.

By examining the unique aphid inclusions in Canadian amber—the youngest of the Cretaceous ambers—we fill gaps in our knowledge regarding the faunal turnover at the Mesozoic–Cenozoic boundary. We estimate the rates at which “aphid vivipara” groups emerged and “aphid ovipara” groups went extinct.

In addition to describing new species, we discuss the current knowledge of taxonomic and morphological diversity of the aphids in Canadian amber. We examine the affinities and differences among Late Cretaceous aphid fauna on a global scale. We also compare selected morphological structures between the Late Cretaceous and the Cenozoic aphids.

## 2. Materials and Methods

The newly described fossil specimens, as well as examined holotypes, are housed in the Harvard Museum of Comparative Zoology (MCZ), Cambridge, USA, Carpenter collection (F.M.C. Coll.). The specimens originate from Cedar Lake, Manitoba. All newly described specimens are preserved in small, polished blocks of amber embedded in artificial resin. The specimens are labelled with the numbers 6851, 7065, 7072 and 7086.

**Figure 2:**
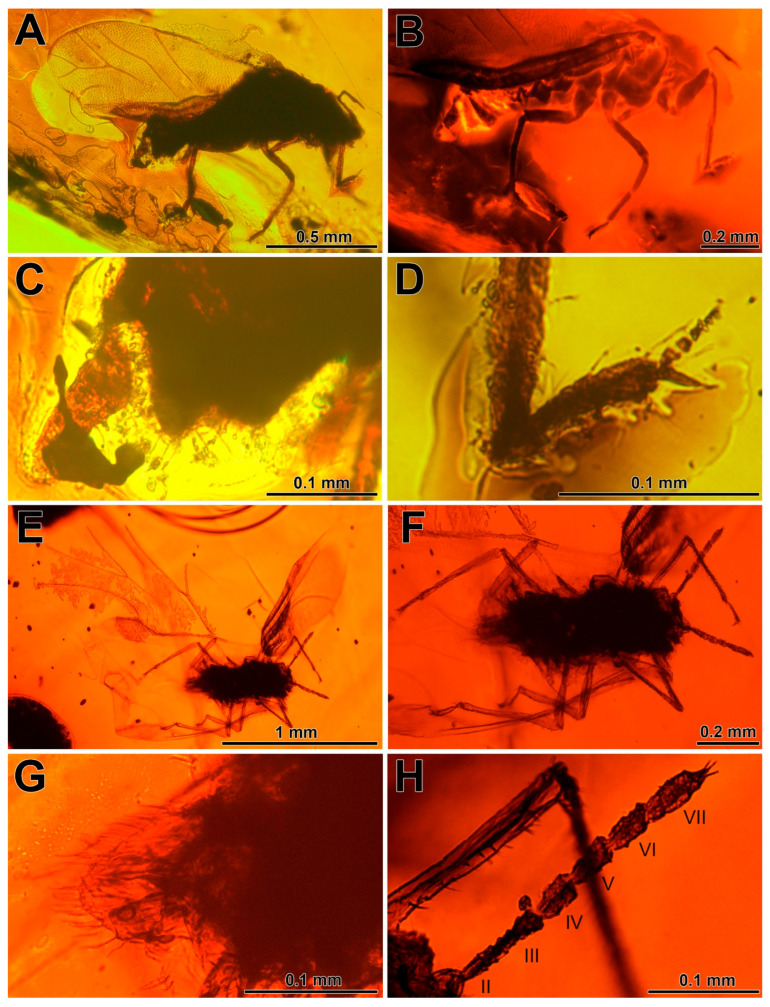
*Alloambria infelicis* holotype: (**A**) lateral side of the body (phase-contrast microscopy), (**B**) lateral side of the body under fluorescence microscope, (**C**) apical part of the abdomen (phase-contrast microscopy), (**D**) fore tarsus; *Ambaraphis kotejai* holotype: (**E**,**F**) dorsal side of the body (fluorescence and phase-contrast microscopy), (**G**) apical part of the abdomen (fluorescence and phase-contrast microscopy), (**H**) left antenna (fluorescence and phase-contrast microscopy). Abbreviations: I–VII antennal segments.

We also analysed holotype specimens of the following species described by Richards [10]: *Palaeoaphis archimedia*, *Ambaraphis costalis*, *Pseudambria longirostris*, *Alloambria caudata* (Figure 1) and by Kania and Wegierek 2005: *Alloambria infelicis* and *Ambaraphis kotejai* (Figure 2). Holotypes of *Alloambria infelicis* and *Ambaraphis kotejai* are preserved in small, polished blocks of amber embedded in artificial resin. Holotypes of *Ambaraphis costalis*, *Pseudambria longirostris* and *Alloambria caudata* are preserved in oil and stored in tightly sealed glass vials.

Specimen 6851 preserves the entire body and is the only alate aphid among the new material. Fore and hind wings are preserved on one side of the body, along with the middle and hind legs and partially preserved antennae. The remaining new specimens are apterous. Specimen 7065 also preserves the entire body, but it is highly obscured. All legs are preserved, together with one fully and one partially preserved antenna, while the rostrum is poorly visible. Specimen 7072 preserves the entire body and all appendages but is darkly preserved, limiting visibility of fine morphological details. Specimen 7086 has all appendages well-preserved and clearly visible.

The specimens were polished to improve observation. They were observed under a Leica M205C stereomicroscope (Leica Microsystems, Wetzlar, Germany) and a Nikon Eclipse-E600 light microscope (Nikon Instruments Inc., Tokyo, Japan) at the Institute of Biology, Biotechnology and Environmental Protection, Faculty of Natural Sciences, University of Silesia in Katowice, Katowice, Poland, as well as under a Nikon Eclipse Ti inverted microscope equipped with fluorescence and phase contrast, at the Laboratory of Evolutionary Entomology and Museum of Amber Inclusions, Department of Invertebrate Zoology and Parasitology, University of Gdańsk, Gdańsk, Poland. Photographs were taken under a Leica M205C stereomicroscope with a Leica flexcam C3 camera attached (using LAS X software ver. 5.1.0.25593.1) and a Nikon Eclipse Ti inverted microscope equipped with fluorescence and phase contrast with a Nikon Digital Sight DS-Fi2 (using NIS F software ver. 54207). Systematics followed [18]. All measurements are given in millimetres.

## 3. Results

Systematic palaeontology

Order Hemiptera Linnaeus, 1758

Suborder Sternorrhyncha Amyot and Audinet-Serville, 1843

Infraorder Aphidomorpha Becker-Migdisova and Aizenberg, 1962

Superfamily Aphidoidea Latreille, 1802

**Family Canadaphididae Richards, 1966**

*Type genus Canadaphis* Essig, 1937

*Alloambria infelicis* Kania & Wegierek, 2005

(Figure 3A,B)

*Material.* MCZ no. 7065, Carpenter collection, apterous morph.

*Diagnosis*. Antenna 6 segmented, without secondary rhinaria. Abdomen narrowing posteriorly, apical region elongated and blunt. Rostrum reaching mid-coxae.

*Description.* Body elliptical in outline, 1.85 long, at the broadest point 0.72 wide. Head poorly separated from thorax. Abdomen and thorax have same width. Abdomen distinctly narrowing from two-thirds of its length, broad at base at 0.72, with a finger-like apex. Bases of six-segmented, slender antennae widely separated. Antennal segment lengths: I 0.04, II 0.03–0.04, III 0.25, IV 0.08, V 0.15, VIa 0.11, VIb 0.05. The first two flagellar segments covered with sparse setae; on segments V and VI, the setae are denser. Circular rhinarium at base of processus terminalis on segment VI. Rostrum thin, reaching middle coxa, apical segment IV (length 0.10, width at base 0.04). Legs relatively short, their length approximately equal to the body’s width. Fore: femur 0.30–0.36, tibia 0.4, tarsal segment I 0.10–0.11, segment II 0.08; middle: femur 0.29, tibia 0.29, tarsus 0.09; hind: tibia 0.51.

Apically rounded anal plate on ventral side of the body (length 0.03; width 0.03). Ovipositor located at base of anal plate.

*Remarks*. Although the Canadaphididae have been known for quite some time, and representatives of the family are abundant in Canadian amber, an apterous morph was first recorded by Ogłaza and Wegierek [17]. The morph described here is only the second known inclusion of a wingless aphid that shares all characteristic features with the family Canadaphididae (regarding the structure of the antennae and the apical structure of the abdomen). The narrowed part of the abdomen in MCZ No. 7065 has approximately one-sixth of the body length, compared to one-fifth in the previously described apterous morph. The newly described morph differs not only in the position of the abdominal constriction but also in the distinctly different morphology of the antennae. In *Canadaphis carpenteri*, antennal segment III is approximately four times shorter than the combined length of the remaining flagellar segments, whereas in the apterous morph of *Alloambria infelicis* this segment is proportionally much longer, reaching 1.5 times the combined length of segments IV–VI.

The structure of the rostrum has only been described in *Alloambria infelicis* Kania & Wegierek, 2005; *Canadaphis mordvilkoi* Kononova, 1976; and *Canadaphis carpenteri*. The rostrum in the described morph reaches the middle coxa, and the structure of segment IV corresponds to that in *Alloambria infelicis*.

**Family Thelaxidae Baker, 1920**

Genus ***Matronaphis*** Ogłaza and Wegierek gen. nov.

Type species *Matronaphis breviantennata* Ogłaza and Wegierek sp. nov.

*Etymology*. *Matrona* _Latin_ = “matron, respectable woman” and the genus *Aphis*.

*Diagnosis.* Body stout. Only three ommatidia. Five-segmented antenna, short, reaching at most base of forelegs. Rostrum long, reaching base of hind legs. Boundary between head and pronotum indistinct.

*Matronaphis*: urn:lsid:zoobank.org:act:4F70EF1C-4133-4C9B-AD7B-1CEAA6D35DBF

***Matronaphis breviantennata*** Ogłaza and Wegierek sp. nov.

(Figure 3C–E)

*Etymology.* Breviantennata _Latin_—compound adjective: brevi- (brevis) = “short” antennata = “bearing antennae”, referring to reduced antennal length.

*Diagnosis*. As for genus (monotypic).

*Matronaphis breviantennata*: urn:lsid:zoobank.org:act:8F6AE4E2-79EB-46E5-80F2-1B1655C30E6D

*Material*. Holotype MCZ No. 7072, Carpenter collection, apterous morph.

*Description*. Body stout (length 1.6). Abdomen fairly well-developed (width 0.72) accounting for one -third of the body length. Anterior head margin straight. Antenna bare. Antennal segment lengths: I 0.05; II 0.05; III 0.09; IV 0.04, V 0.13–0.14 (Va 0.06 + Vb 0.07–0.08). The terminal segment is the longest and bears a distinct processus terminalis. Last rostral segments sturdy (0.08 and 0.11). Legs slender (fore: coxa 0.06, femur 0.21, tibia 0.23, tarsal segment I 0.03, segment II 0.11; middle: coxa 0.06, femur 0.24, tibia 0.28, tarsal segment I 0.03, segment II 0.11; hind: femur 0.30, tibia 0.30, tarsal segment I 0.03, segment II 0.12). Abdomen oval with a blunt termination (last segment width 0.21). Genital plate semi-circular, anal plate narrow, lateral margins distinctly narrowed.

*Remarks*. Apterous morphs within the oviparous aphid superfamily (Aphidoidea) were previously described from the Late Cretaceous [13,14,17]. Although these fossils are relatively scarce, their morphology proves to be remarkably diverse.

*Sidorchukaphis katyae* Ogłaza and Wegierek, 2024 is the only Late Cretaceous aphid from the “vivipara” group with which the described genus and species can be compared. However, despite sharing several morphological features, including a generally similar body shape with a strongly developed, broad abdomen and thick antennae composed of short segments, these two genera differ markedly in a number of diagnostic characters. *Sidorchukaphis* is distinguished by the head and pronotum being separated, a processus terminalis shorter than the basal part of the last antennal segment, a long rostrum extending to the end of the body, a broadly rounded cauda, and the presence of wax glands.

The fusion of the head and pronotum is characteristic of representatives of the wingless morphs of several extant families: Greenideidae, Hormaphididae, Mindaridae, Phloeomyzidae, and Thelaxidae. These features are absent in the Pemphigidae (Eriosomatidae). Unlike other groups, the Greenideidae have very well-developed siphunculi and eyes. The Mindaridae have six-segmented antennae and a well-defined cauda, while the Phloeomyzidae have a well-developed wax gland, which was not found in *Matronaphis breviantennata*. In the wingless Hormaphididae, we observe that the antennae and legs are poorly developed.

When conducting this type of analysis, *Matronaphis breviantennata* Ogłaza and Wegierek sp. nov. should be assigned to the Thelaxidae (anal plate not bilobed). The lack of knowledge about the shape of the cauda does not preclude *Matronaphis breviantennata* from being classified in this family, as the presented analysis of the features indicates such affiliation. The structure of the cauda is not decisive here; it may vary among genera included in this family, e.g., *Thelaxes*—cauda knobbed, *Glyphina*—cauda semicircular.

Aphidoidea *incertae sedis*

Genus ***Curraphis*** Ogłaza and Wegierek gen. nov.

Type species *Curraphis velox* Ogłaza and Wegierek sp. nov., by present designation.

*Etymology*. *Currens* _Latin_—“running, moving”, participle of the verb *currere* (“to run”) and the genus *Aphis*.

*Diagnosis.* Six-segmented antenna with a well-developed processus terminalis. Flagellar segments III-V with large, round rhinaria. Cauda long, finger-shaped.

*Curraphis*: urn:lsid:zoobank.org:act:4D015714-8B0F-4C5A-9CC3-2DE4B1CB1C51

***Curraphis velox*** Ogłaza and Wegierek sp. nov.

(Figure 4A–E)

*Etymology.* Velox _Latin_ (“fast”)

*Diagnosis*. As for genus (monotypic).

*Curraphis velox*: urn:lsid:zoobank.org:act:7D799BFD-43CE-418F-A9DE-09AEADE9B12F

*Material*. Holotype MCZ no. 7086, F.M.C. coll., apterous morph.

*Description*. Body slender (0.95). Antennal segment lengths: I 0.03, II 0.03, III 0.16, IV 0.09, V 0.11, VI 0.12 (VIa 0.08 + VIb 0.04), reaching half the length of the abdomen. Processus terminalis approximately half as long as segment VIa. Flagellar segments with sparse, single setae. Rhinaria large, with a diameter equal to that of individual antennal segments. Rostrum reaching behind the base of hind legs, rostral segment III 0.04, segment IV twice as long (0.08) as wide, apical part of the last segment, with two bristles equal in length or longer than the segment width. Apex of rostrum blunt. Legs slender (fore: tibia 0.26, tarsal segment I 0.02, segment II 0.08; hind: tibia 0.41, tarsal segment I 0.02, segment II 0.07), femur, tibia and tarsus of all pairs covered with dense setae. Apical part of abdomen bulbous, covered with abundant, strong setae. Cauda arising from posterior abdominal apex (caudal base 0.03 wide, 0.1 long). Cauda covered with numerous setae as long as caudal base width.

*Remarks*. Among the aphids in Canadian amber, Canadaphididae have a well-developed cauda, similar to that in *Curraphis velox* Ogłaza and Wegierek sp. nov. [17,19]. Unlike the newly described species, the representatives of Canadaphididae lack rhinaria on individual flagellar segments.

*Curraphis velox* Ogłaza and Wegierek sp. nov. shows morphological similarity to *Aphidocallis caudatus* Kononova, 1977 in the shape of the cauda and the pattern of rhinaria distribution. However, the species described by Kononova [20] is an alate morph with five-segmented antennae, sparsely distributed rhinaria and a short processus terminalis. It originates from older (Santonian) Taimyr amber [21].

The detailed examination of the morphology of *Curraphis velox* revealed no clearly developed siphunculi. In Aphididae, siphuncular structures vary considerably, ranging from well-developed tubular forms to highly reduced or pore-like conditions; however, in the present fossil inclusion, preservation does not allow confident assessment of their presence or absence. Furthermore, the antennal morphology, particularly the elongated processus terminalis, is consistent with that of representatives of Aphididae. Kononova [20] assigned *Aphidocallis* to the family Aphididae. Nevertheless, owing to the incomplete preservation of diagnostic morphological characters in specimen MCZ No. 7086, *Curraphis velox* cannot be confidently assigned to a specific aphid family.

Superfamily Palaeoaphidoidea Richards, 1966

Family **Palaeoaphididae** Richards, 1966

Subfamily Palaeoaphidinae Richards, 1966

Type genus. *Palaeoaphis* Richards, 1966, by original designation and monotypy.

Genus ***Latialiaphis*** Ogłaza and Wegierek gen. nov.

Type species *Latialiaphis oblita* Ogłaza and Wegierek sp. nov., by present designation.

*Etymology*. *Latis alis* _Latin_—“with broad wings” and the genus *Aphis.*

*Diagnosis*. Forewings broad, approximately half as wide as wing length. Veins Rs and M_1+2_ as well as M_3+4_ sinusoidally curved, not straight.

*Latialiaphis*: urn:lsid:zoobank.org:act:C8756DFA-485E-4342-AAC8-A2FD66EAA5EC

***Latialiaphis oblita*** Ogłaza and Wegierek sp. nov.

(Figure 4F–I)

*Etymology*. Oblita _Latin_—“forgotten” (feminine form, matching the grammatical gender *Aphis*).

*Diagnosis.* As for the genus (monotypic).

*Latialiaphis oblita*: urn:lsid:zoobank.org:act:CEA5F41B-AC35-4D42-9854-5EE5DB3AA811

*Material.* Holotype MCZ no. 6851, Carpenter collection, alate morph.

*Description*. Body slender (length 0.92). Head distinctly separated from thorax. Antenna seven-segmented (VI 0.08, VII 0.09). The last antennal segment gradually tapering from halfway its length towards a sharp apex. Segment VI with five and segment VII with six annular rhinaria. Legs slender (middle: tibia 0.38–0.41, tarsal segment I 0.02, segment II 0.10; hind: femur 0.22, tibia 0.33–0.37, tarsal segment I 0.02, segment II 0.09–0.10.), tibia and tarsus covered with abundant, stout setae. Forewings spatulate (length 1.3, width 0.81) broadest at the pterostigma base (0.81). Wing (length 1.3, width 0.81) membranes and Pt covered with scale-shaped setae, Pt lenticular in shape (length 0.36, width 0.13). Main stem of CuA very short, forming one line with CuA_1_. Vein M elongation reaching main stem in the middle of its length, between CuA and Pt bases. M broadly branched. Both M_1+2_ and M_3+4_ curved, sinusoidally curved Rs separating halfway of the Pt length. Base of abdomen as wide as thorax (0.19). Individual abdominal segments clearly outlined, apical part of abdomen (0.07) distinctly narrowed, finger-shaped.

*Remarks.* Rs arises at the base of pterostigma in three known species (*Longiradius foottitti* Heie, 2006; *Palaeoaphidiella abdominalis* Heie 1996 and *Palaeoaphis armani* Wegierek, 1993) belonging to the subfamily Palaeoaphidinae.

The remaining species, including *Latialiaphis oblita* Ogłaza and Wegierek sp. nov., have Rs separating from Pt approximately halfway along its length. In *Ambaraphis costalis* Richards, 1966; *Ambaraphis kotejai* Kania & Wegierek, 2005; *Ambaraphis baikurensis* Wegierek and Perkovsky, 2017; *Palaeoaphis archimedia* Richards, 1966; and *Palaeoaphis incognita* Kononova, 1976 the base of vein M is directed towards CuA base. The newly described genus and species seem to be exceptions to this rule, because M is directed towards the common stem Sc + R + M. The shape of the wing in *Latialiaphis oblita* Ogłaza and Wegierek sp. nov. also appears unique within the subfamily. Although atypical in form, the wings are not deformed. This is indicated by regular courses of veins and the same location in relation to Pt and common stem Sc + R + M.

## 4. Discussion

The current list of described aphid taxa from Canadian amber has been supplemented with new species belonging to previously described families: the extinct family Palaeoaphididae (*Latialiaphis oblita* Ogłaza and Wegierek sp. nov.) and the extant family Thelaxidae (*Matronaphis breviantennata* Ogłaza and Wegierek sp. nov.). Additionally, a wingless morph has been described for the first time within the previously described species *Alloambria infelicis*. The wingless morph of *Curraphis velox* Ogłaza and Wegierek sp. nov. has not been clearly assigned to a specific family due to the lack of clear taxonomic characters, although several features indicate a close relationship to Aphididae.

The current known species composition of aphids from Canadian amber includes 7 families, 19 genera, and 24 species (including the taxa described in this article). Most species are known only from this resin, with the exception of *Cretamyzus pikei* Heie, 1992, which was described from a large piece of amber associated with a hadrosaurian dinosaur specimen [14].

To date, only representatives of two extinct families have been described from Canadian amber: Canadaphididae (four species, including one wingless morph) and Cretamyzidae (a single wingless morph), belonging to the superfamily Aphidoidea of viviparous aphids. Our research, based largely on wingless morphs, supplements this very limited information.

For the first time, species assignable to extant aphid families have been reported from Canadian amber. Thus, alongside Taymyr amber, this amber represents an important source of information on the early evolution of the dominant aphid families from the group Vivipara (over 5000 species; Ovipara approximately 150 species) in modern fauna.

Similar to Canadian amber (which is the youngest known resin of the epoch to contain aphid inclusions), Taimyr amber contains abundant aphid fossils. Burmese amber (Cenomanian, Early Late Cretaceous) is among the oldest amber deposits in which aphids are commonly reported [22]. In Early Cretaceous insect-bearing deposits, aphids are predominantly known from compression fossils preserved as impressions in sedimentary rocks. Only a limited number of localities yielding aphid compression fossils are currently known. Among them are Orapa (Turonian) in the Orapa Diamond Mine, Botswana [23], and Obeshchayushchiy (Santonian–lower Campanian), north-eastern Russia, Magadan Oblast [24].

The aphid fauna in Burmese amber is highly endemic and shows no clearly shared taxa with other Late Cretaceous assemblages [25,26,27,28]. The fauna of Gondwanan origin in Orapa has yielded only two alate specimens of aphids, but their taxonomic position remains uncertain [29].

All Late Cretaceous localities of fossil aphids in the Northern Hemisphere have yielded genera and species assignable to the families Canadaphididae and Palaeoaphididae.

The Obeshchayushchiy locality is notable for the reported presence of Oviparosiphidae Shaposhnikov, 1979. Previously, this family had been described primarily from Late Jurassic and Early Cretaceous deposits in Russia, China, and Mongolia [30]. The absence of Tajmyraphididae Kononova, 1975 also seems noteworthy, as they are common among Taimyr amber inclusions [21] and have been recorded in Canadian amber as well [12].

The abundance of alate morphs belonging to the families Canadaphididae and Palaeoaphididae is higher in Canadian amber (17 specimens) than in Taimyr amber (7 specimens) [21]. Canadian amber may also contain a higher proportion of larval inclusions, although the exact number is not known [9]. However, Taimyr amber inclusions are taxonomically more diverse than those from Canadian amber, except for taxa representing extinct families (Canadaphididae, Elektraphididae, Palaeoaphididae, Shaposhnikovidae and Tajmyraphididae). Taimyr amber is also the source of several of the first-described earliest representatives of several extant families [21].

Even though Canadian amber is much younger (about 10 million years old) than Taimyr amber, aphids attributable to extant families appear to be less frequently represented in Canadian material. This pattern may reflect differences in paleoenvironmental conditions.

Our study was based on only a limited faunal sample due to taphonomic factors and selective preservation in the fossil record. More information could be obtained by comparing the aphid fauna from Canadian amber with other sites from the same period using ecological data and advanced statistical methods. This would provide additional insights into the aphid fauna of the Late Cretaceous and its diversity.

Next, the described materials, along with other data from the Late Cretaceous, will be used for comparisons with Eocene aphid fauna, for example, from Sakhalin amber.

The significance of this research on the Cretaceous-Cenozoic transition in aphid history lies in the fact that the Late Cretaceous saw the emergence of taxa representing modern families while being a time when representatives of the oldest families—such as the Oviparosiphidae—coexisted with families known only from the Late Cretaceous.

These findings provide tangible evidence that the origins of modern families can be traced to the Cretaceous–Cenozoic boundary, a conclusion that contrasts the molecular estimates.

## 5. Conclusions

In contrast to the Jurassic–Early Cretaceous interval of aphid evolution, the current knowledge of aphid fauna in the Late Cretaceous relies almost exclusively on inclusions preserved in fossil resins. Alate morphs remain predominant in Late Cretaceous aphid assemblages, whereas sexually mature apterous morphs are found only sporadically (e.g., Taimyr and Canadian amber).

Late Cretaceous aphid faunas include both endemic extinct families (Cretamyzidae, Grassyaphididae, Isolitaphidae, Mesozoicaphididae, Parvaverrucosidae, Shaposhnikovidae) and taxa assigned to extant families, such as Aphididae, Drepanosiphidae and Eriosomatidae.

Apterous morphs described from Late Cretaceous deposits share several morphological features with extant aphids, including antennae with distinct processus terminalis, caudal structures, single secondary rhinaria and wax glands on the abdomen. Therefore, Canadian amber represents an important source of information on the morphological diversity and evolutionary history of Late Cretaceous aphids.

## Figures and Tables

**Figure 3:**
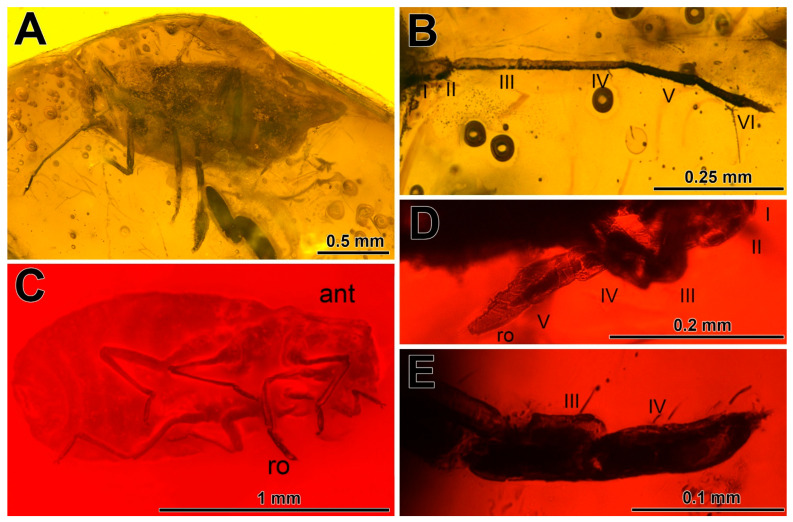
*Alloambria infelicis* (specimen 7065): (**A**) ventral side of the body (light microscopy), (**B**) right antenna (light microscopy); *Matronaphis breviantennata* holotype: (**C**) ventral side of the body (fluorescence and phase-contrast microscopy), (**D**) right antenna (fluorescence and phase-contrast microscopy), (**E**) apical part of the rostrum (fluorescence and phase-contrast microscopy).

**Figure 4:**
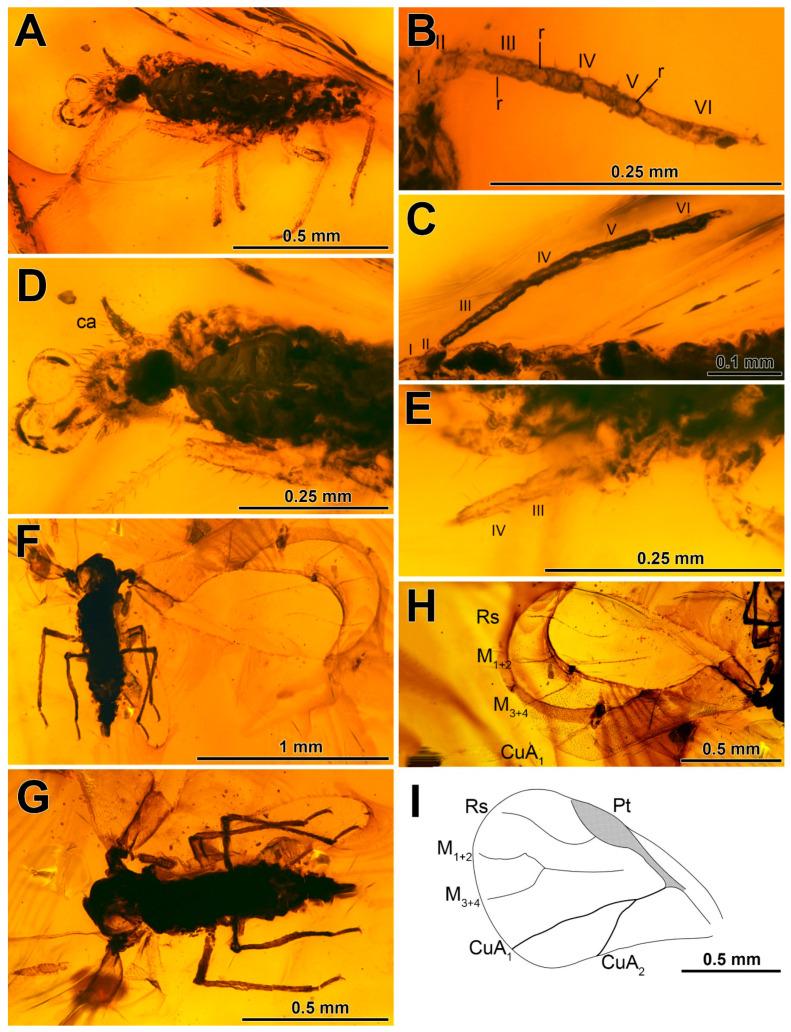
*Curraphis velox* holotype: (**A**) lateral side of the body (light microscopy), (**B**) right antenna (light microscopy), (**C**) left antenna (light microscopy), (**D**) apical part of the abdomen (light microscopy), (**E**) apical part of the rostrum (light microscopy). *Latialiaphis oblita* holotype: (**F**,**G**) dorsal side of the body (light microscopy), (**H**) forewing (light microscopy), (**I**) reconstruction of wing. Abbreviations: ca—cauda, r—rhinaria; I–VI—antennal segments, Pt—pterostigma, Rs—radial sector, vein M—media vein, CuA_1_, CuA_2_—cubital veins.

## Data Availability

The original contributions presented in this study are included in this article. Further inquiries can be directed to the corresponding authors.

## Abbreviations

The following abbreviations are used in this manuscript:

MCZ	Harvard Museum of Comparative Zoology
